# Improved Resolution Haplogroup G Phylogeny in the Y Chromosome, Revealed by a Set of Newly Characterized SNPs

**DOI:** 10.1371/journal.pone.0005792

**Published:** 2009-06-04

**Authors:** Lynn M. Sims, Dennis Garvey, Jack Ballantyne

**Affiliations:** 1 Biomedical Sciences, Graduate Program in Chemistry, University of Central Florida, Orlando, Florida, United States of America; 2 Department of Chemistry, University of Central Florida, Orlando, Florida, United States of America; 3 National Center for Forensic Science, Orlando, Florida, United States of America; 4 Y Line Genetics, Spokane, Washington, United States of America; Louisiana State University, United States of America

## Abstract

**Background:**

Y-SNP haplogroup G (hgG), defined by Y-SNP marker M201, is relatively uncommon in the United States general population, with only 8 additional sub-markers characterized. Many of the previously described eight sub-markers are either very rare (2–4%) or do not distinguish between major populations within this hg. In fact, prior to the current study, only 2% of our reference Caucasian population belonged to hgG and all of these individuals were in sub-haplogroup G2a, defined by P15. Additional Y-SNPs are needed in order to differentiate between individuals within this haplogroup.

**Principal Findings:**

In this work we have investigated whether we could differentiate between a population of 63 hgG individuals using previously uncharacterized Y-SNPs. We have designed assays to test these individuals using all known hgG SNPs (n = 9) and an additional 16 unreported/undefined Y-SNPS. Using a combination of DNA sequence and genetic genealogy databases, we have uncovered a total of 15 new hgG SNPs that had been previously reported but not phylogenetically characterized. Ten of the new Y-SNPs are phylogenetically equivalent to M201, one is equivalent to P15 and, interestingly, four create new, separate haplogroups. Three of the latter are more common than many of the previously defined Y-SNPs. Y-STR data from these individuals show that DYS385*12 is present in (70%) of G2a3b1-U13 individuals while only 4% of non-G2a3b1-U13 individuals posses the DYS385*12 allele.

**Conclusions:**

This study uncovered several previously undefined Y-SNPs by using data from several database sources. The new Y-SNPs revealed in this paper will be of importance to those with research interests in population biology and human evolution.

## Introduction

Single nucleotide polymorphisms (SNPs) are the smallest and most abundant type of human DNA polymorphisms [1]. SNPs have been extensively used in the study of human evolutionary and migratory patterns [2] and are increasingly being used in genome-wide association studies [3]. Y-SNPs, in particular, are of interest due to their paternal inheritance, lack of recombination, abundance, and low mutation rate and are currently being investigated for characterizing male population structure and ethnogeographic origin in forensic science [4]–[10]. These unique polymorphisms within the non-recombining region (NRY) of the Y-chromosome (mainly SNPs) have created population specific paternal lineages (commonly called haplogroups) that have persisted throughout human history. Large scale parsimonious phylogenetic trees representing world wide Y chromosomal variation have been constructed and comprise the major haplogroups A-T [11]–[13]. The rules for naming haplogroups have been designed to adjust for new SNPs that are continuously being identified and characterized to be added to the tree and potentially reshaping it as in the most recently published y-chromosomal haplogroup tree [13]. Although some are rare (e.g. [14]), some can still be useful for individual identification especially if found at higher frequencies in certain defined populations. Many of these polymorphisms have proven highly informative in tracing human prehistoric migrations and generating new hypotheses on human colonization and migrations [15].

This study recruited 54 hgG men from the pool available in Ysearch.org (http://www.ysearch.org) in addition to 9 that we possessed in-house. We have uncovered a total of 15 new hgG SNPs, four of which create new sub-haplogroups with the hgG clade. Additionally, we have also discovered that the Y-STR DYS385*12 is present in (70%) of the new hg G2a3b1-U13 individuals and only 4% of non-G2a3b1-U13 individuals.

## Results

**Figure 1:**
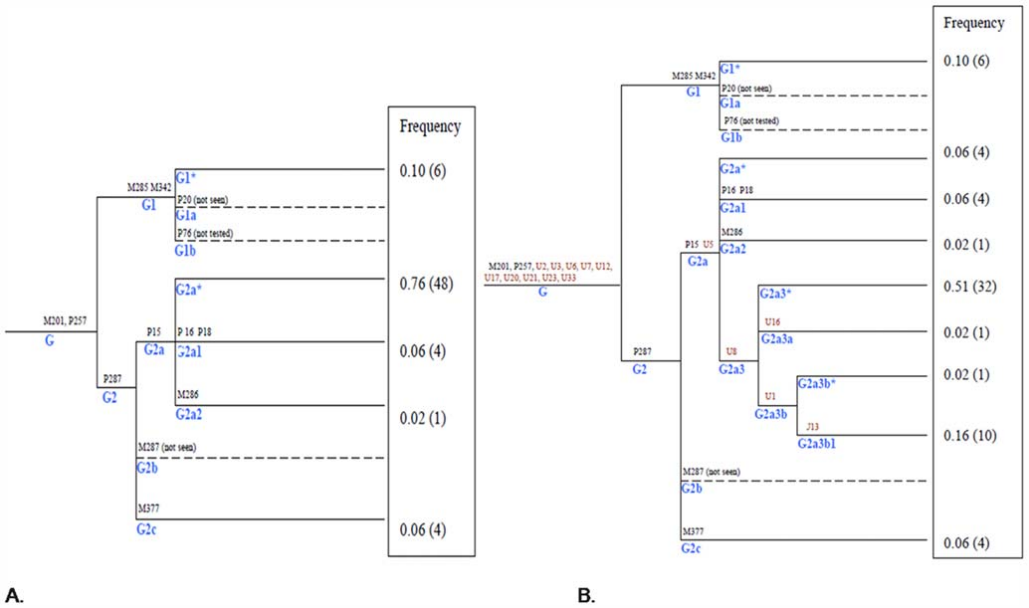
Phylogenetic Trees Indicating the Frequencies of Haplogroup G Individuals with (B) and without (A) the Newly Characterized Y-SNPs. The haplogroup names are assigned base on the most recent haplogroup tree by Karafet et al. [13].

Phylogenetic trees were constructed to show the evolutionary relationships between the previously characterized hgG SNPs and 15 newly characterized hgG SNPs (Figure 1A and 1B). The population frequencies of Haplogroup G individuals in sub hgG groups without (Figure 1A) and with the newly characterized Y-SNPs are also shown (Figure 1B). Eleven of the new Y-SNPs were phylogenetically indistinct from the current G-M201 and G2a-P15 markers. However, four newly characterized SNPs (U8, U16, U1, U13) permitted the definition of four new sub-clades of hg G (G2a3*, G2a3a, G2a3b*, G2a3b1) which, in our population sample, increased the number of observable hg G genotypes by 80% (from five to nine). To ascertain the extent to which the new markers are useful for differentiating hgG individuals, the probability of discrimination (DP) obtained by typing individuals with and without the four new informative markers was calculated [16]. The DP was increased 72%, from 0.40 to 0.69, in our Caucasian sample set.

Examination of the allele distribution of DYS385 showed that the allele DYS385*12 was over-represented in the G2a3b1-U13 samples (7/10 samples possess the 12 allele). Only two copies of the DYS385*12 allele were found in the other 53 non-G2a3b1-U13 samples (4%). Thus the DYS385*12 is highly predictive (P = 0.70) of a G2a3b1-U13 individual if the individual is known to belong to hgG. Such Y-STR information can sometimes facilitate a speedier haplogroup assignment and differentiation than would be available from a strict hierarchy-based SNP analysis.

In summary, we have characterized 15 new hgG SNPs that had been previously reported but not phylogenetically defined. Ten of the new Y-SNPs are phylogenetically indistinguishable from G-M201, one is equivalent to G2a-P15 but four create new, separate hg G sub-haplogroups. Three of the latter are more common than many of the previously defined hgG Y-SNPs.

## Discussion

Y-SNP haplogroup G (hgG), defined by the Y-SNP marker G-M201, is relatively uncommon in the European American population of the United States. This haplogroup is thought to have originated in the Caucasus region of Eurasia, especially in the North Ossetians [17] and specifically, the Digora population with an average frequency of 74% [18]. Also approximately 11% of individuals in Anatolia [19] and 17% in Northern Sardenia [20] belong to hgG. In a study on Y-chromosomes in the Caucasus, it was found that the hgG genotype frequency ranges from 21%–74% in seven different populations of the north Caucasus region while it is only found within 3 populations in south Caucasus region (at frequencies of 11%, 18%, and 31%) [18]. It has been proposed that the peoples from these regions originated from West Asia rather than Europe due the high frequencies of the G, J2* and F* haplogroups [17].

Prior to this study, only 8 sub-markers had been described within haplogroup G[11], [19], [21] with the most common haplogroup being G2a*, defined by P15. Many of the previously described eight sub-markers are either very rare or do not distinguish between major populations within this haplogroup. In fact, prior to the current study, only 2% of our reference European American population was within hgG and all of the individuals were in sub-haplogroup G2a defined by the P15 polymorphism [10]. In this work we have investigated whether we could differentiate between a population of 63 hgG individuals using previously uncharacterized Y-SNPs as well as their associated 19 marker Y-STR haplotypes. Here we describe the characterization of new hgG sub-markers, four of which can further differentiate between sub-populations within this hg.

The subjects were recruited from a selection of over 500 haplogroup G men available in public genetic-genealogy databases in the fall of 2006. This was especially valuable in the case of a somewhat rare European haplogroup like haplogroup G (about 2–4% of the general population) [10], [19], [22], [23]. The selection of hgG men from already SNP typed populations provided a tremendous savings in time and cost for this project. To have found this large of a sample of HgG men without the resource of the genetic genealogy community's results would have necessitated the SNP typing of approximately 1500 men. This study exemplifies the success obtainable by productive collaboration between genetics researchers and the genetic genealogy community.

## Materials and Methods

This study was conducted according to the principles expressed in the Declaration of Helsinki. The study was approved by the Institutional Review Board of the University of Central Florida. All participants provided written informed consent for the collection of samples and subsequent analysis.

### Candidate SNP Identification

DNA sequence traces from the NCBI Trace Archives were used to identify SNPs of an individual from haplogroup G, *in silico*. Most of the earliest mapping of the Y chromosome was done using the BAC library from the California Institute of Technology called CTC. This included the contigs: AC005942.2 CTC-298B15, AC002992.1 CTC-203M13, AC004617.2 CTC-264M20, AC002531.1 CTC-486O8, AC004474.1 CTC-475I1, AC006565.4 CTC-484O7, AC005820.1 CTC-494G17, and AC078938.3 CTC-480L15. The Y-SNP marker M201 in contig AC004474 was seen to be derived rather than ancestral in the reference sequence. This means that the man used in the CTC library belonged to YCC haplogroup G. The assumption was made that the contents of the NCBI Trace Archives were not likely to contain the re-sequencing of any other haplogroup G men (since G is seen in only about 3% of the males of European descent). Candidate YSNPs were chosen on the basis of appearing in only the above reference contigs and none of the traces in the Trace Archives. Those candidate SNPs were typed and characterized in a panel of 63 haplogroup G men. Additionally, all previously defined hgG Y-SNPs were compiled from various publications and a 25 member candidate list of SNPs suspected to be polymorphic inside haplogroup G was developed for assay development and subsequent population studies.

### Biological Sample Donors

Buccal swabs were obtained from a total of 74 individuals including 63 human males belonging to Y-SNP hgG, one human female, one male chimp, and one human individual from each of the following hgs: A, B, C, E3a, F, H, I, J, and R1b. Fifty-four of the individuals were recruited from the list of individuals whose 37 locus Y-STR haplotypes and Y-SNP haplogroups are available in http://www.ysearch.org. All DNA samples were obtained with the individual's informed consent in accordance with the University of Central Florida's Institutional Review Board.

### Genomic DNA isolation

A small piece of the buccal swab was cut and placed in a Spin-ease tube (Gibco-BRL, Grand Island, NY) and incubated overnight at 56°C in 400 µL DNA extraction buffer (100 mM NaCl, 10 mM Tris-HCl, pH 8.0, 25 mM EDTA, 0.5% SDS, and 0.1 mg/ml Proteinase K). The cut material was then removed from the tube and placed in a Spin-ease basket and the basket was then placed back in the original tube and centrifuged (Eppendorf Centrifuge 5415D) at 13,200 rpm for 5 min. The extract was then purified and isolated using 25∶24∶1 phenol/chloroform/isoamyl achohol (Fisher Scientific, Norcross, GA) followed by filtration using Microcon® 100 centrifugal filter devices (Millipore, Bedford, MA) according to the manufacturer's instructions. Samples were brought to a final volume of 50 µL in TE^−4^ (10 mM Tris-HCl and 0.1 mM EDTA, pH 7.5) and stored at 4°C until analysis. DNA was quantified by gel electrophoresis in a 1% Agarose gel. Samples were visualized using the ΩMega 12*ic*™ Gel documentation system (Ultra-Lum, Claremont, CA). Quantification was accomplished by a comparison of the fluorescence intensity of the unknown bands to a set of known quantity standards that were run simultaneously.

### SNP Primer Design and PCR

Assays were developed to amplify regions flanking the SNP for use with pyrosequencing technology. Extracted female DNA was also tested to ensure male specificity. All SNPs were tested against individuals from hgs A, B, C, E, F, H, I, J, and R for haplogroup determination and ancestral vs. derived states. A male chimp was also tested to facilitate determination of the ancestral vs. derived states. PCR primers were designed using a combination of Primer3[24] and SNP Primer Design Pyrosequencing AB v.1.0.1.software. The 50 µL PCR reaction contained: 1 ng DNA, 0.2 µM each primer, 125 µM dNTPs, 1X PCR Buffer II (10 mM Tris-HCl, pH 8.3, 50 mM KCl), 2.0 mM MgCl2, 10 µg non-acetylated BSA (Sigma, St. Louis, MO) and 1.5 units of AmpliTaq® Gold Polymerase (Applied Biosystems, Foster City, CA). Cycling conditions were: (1) 95°C for 10 min, (2) 40 cycles: 95°C for 15 s, 54°C for 30 s, 72°C for 15 s, and (3) final extension at 72°C for 5 min, unless otherwise noted.

Table 1 provides details of the SNP loci used and the PCR primers used.

**Table 1:** List of Y-SNPS, their associated database references, and location of the SNP within the product for the indicated primers.

SNP Name	rs # or AC#: SNP†	Product Size (bp)	Position (bp)	Forward (5′-3′)	Reverse (5′-3′)
**M201**	AC004474.1: G>T	310	199	CATGGGTAATTCGGTTGTTACC	GCCCTTTGGTGGCATAGTA
**P15**	AC007876.2: C>T	75	34	GAATAGAGCCAATGCTTGAGGT	TATGGGAATCACTTTTGCAACT
**P16**	AC068541.7: A>T	84	34	GTCGTTTATTTGGTGCCTGAA	CTATGACCTCAGCAGAATGGA
**P18**	AC016698.3: C>T	141	80	GGTTGGGATTGTGACTCCTCT	CTGGGCAAATTTACCTGTCTC
**P20**	AC016698.3: C>del	141	30	GGTTGGGATTGTGACTCCTCT	CTGGGCAAATTTACCTGTCTC
**M285**	AC007678.3: C>G	188	63	GAGCCGTTGTCCCTGTGTTT	TGCAGGCATCAGCTAGATTGT
**M287**	AC007678.3: A>T	188	93	GAGCCGTTGTCCCTGTGTTT	TGCAGGCATCAGCTAGATTGT
**M286**	AC007678.3: G>A	188	123	GAGCCGTTGTCCCTGTGTTT	TGCAGGCATCAGCTAGATTGT
**M342**	AC007876.2: C>T	103	40	AACAGGGCGTAACAAATAGGT	TGGCTCTATTCATGTGAGGAA
**M377**	AC004474.1: A>G	195	93	TCGGTTGTTACCTTGAGCATT	CGAAAAACCTCAGTTGATACTGG
**U1**	rs9785956: A>G	189	118	TTTCTGCTCCAAATCTGCTG	CACCTGTAATCGGGAGGCTA
**U2**	rs9786712: G>A	150	123	AGCTCATCTTCACGGGTGTG	ACAGGGCAAAGGAATCGTTA
**U3**	rs11799152: A>G	153	45	CTTGAACCCAAGACGAGGAG	CAACAGTGGATCCCACATCA
**U6**	rs2740980: G>A	71	43	CCTCAGTCTCTCCGATTCCTT	CTTTCATCTCCAACCCCCATC
**U5**	rs2178500: T>G	64	24	CTATCACCCAGAGACCCCTCA	GAATCGGGTCCCATAACAAT
**U7**	rs7067251: G>C	222	124	GATCCCACAGAGTGCTCAGG	CATGGGGTAAGAGAATGGGTA
**U8**	rs7892988: T>C	70	45	TATAACCAAAAATGGCACGAT	GGATTAAGGTTGCCATCAGG
**U12**	rs9786640: A>C	162	43	CTATGAGCATTTGGGGGCTTA	CCTTGTATCCTCCCTCCCTTT
**U13**	rs9786706: C>T	81	48	GTGGTAACAGCTCCTGGTGAG	TGCTGCTTTGGTTAACTGTCC
**U16**	rs35474563: C>T	107	30	CTCATTTGACTTCCCGCTGT	CTAGGACGCAGACGTCTTACC
**U17**	rs34742138: C>T	175	150	CAGTTGTGCATCAACCATTCA	AACCTAGGTACTTCTTCCACTTCTC
**U21**	AC005820.1: C>T	98	53	CTGGCACCTCCTCTCACTTC	TCAAGGAGCTTCCACTCACG
**U20** *	rs16648: AG>del	119	61	AGACAAAGTCGGGGTTTTGA	GATCTGCCTCTTTCCCAAAAT
**U23** *	rs9786460: G>A	181	58	GGTGGGAGAATTACCTGAACC	AGCTTCCTGAGTTCCCTTTTT
**U33** *	rs1125978: C>G	94	68	CCCAATGTCCCTCTTTCCTT	ACCCACTTTGACCCAATCTG

† SNP is listed as: ancestral>derived.

* U20 and U33 were amplified at 56°C annealing temperature and U23 was amplified at 57°C for only 35 cycles.

### SNP/STR Genotyping and Phylogenetic Analysis

SNP genotyping was performed by pyrosequencing on a PSQ™ 96 MA instrument according to the manufacturer’s recommendations (Biotage, Uppsala, Sweden, http://www.biotage.com). SNP genotype data were collected and the phylogenetic relationships were depicted in a phylogenetic tree showing the corresponding frequencies for each haplogroup observed. Haplogroups were assigned based on the most recent comprehensive Y chromosomal haplogroup tree published by Karafet et al. [13]. All STR genotyping (19 locus haplotypes) was performed on a Macintosh-based ABI Prism 310 capillary electrophoresis system using two validated multiplex systems, MP I and MP II, as previously described [25], [26]. For two individuals whose STR haplotypes were not determined as just described, some Y STR data were available from http://www.ysearch.org. The probability of discrimination (DP)[16] was calculated as: DP = 1−∑p*_i_* ^2^, where p*_i_* is the observed frequency of the derived allele at each of the sub hg G haplogroups.
